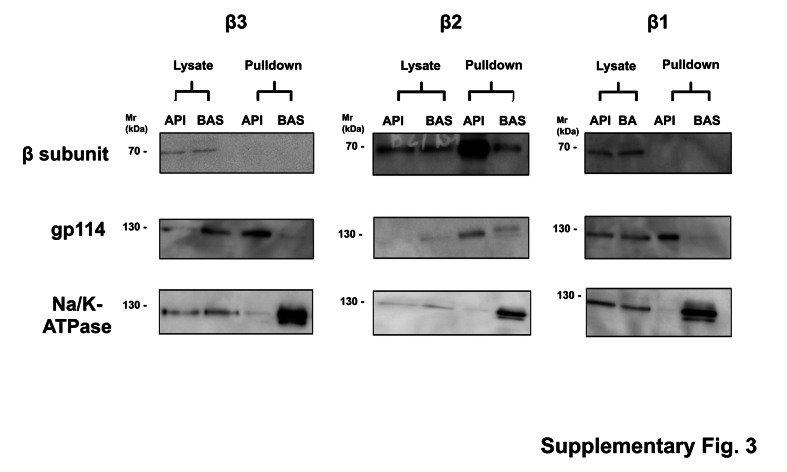# Role of protein domains in trafficking and localization of the voltage-gated sodium channel β2 subunit

**DOI:** 10.1016/j.jbc.2024.107833

**Published:** 2024-09-28

**Authors:** Eric Cortada, Ramon Brugada, Marcel Verges

**Affiliations:** 1 Cardiovascular Genetics Group, Girona Biomedical Research Institute (IDIBGI-CERCA), Edifici IDIBGI, Salt, Province of Girona, Spain; 2 Biomedical Research Networking Center on Cardiovascular Diseases (CIBERCV), Salt, Province of Girona, Spain; 3 Department of Medical Sciences, University of Girona Medical School, Girona, Spain; 4 Department of Cardiology, Hospital Josep Trueta, University of Girona, Girona, Spain

**Keywords:** cell culture, protein trafficking (Golgi), cell polarity, sodium channel, glycosylation, MDCK cells, voltage-gated sodium channel, *SCN2B*

## Abstract

The voltage-gated sodium (Na_V_) channel is critical for cardiomyocyte function since it is responsible for action potential initiation and its propagation throughout the cell. It consists of a protein complex made of a pore forming α subunit and associated β subunits, which regulate α subunit function and subcellular localization. We previously showed the implication of *N*-linked glycosylation and *S*-acylation of β2 in its polarized trafficking. Here, we present evidence of β2 dimerization. Moreover, we demonstrate the implication of the cytoplasmic tail, extracellular loop, and transmembrane domain on proper β2 folding and export to the cell surface of polarized Madin-Darby canine kidney cells. Substantial alteration, or lack of any of these domains, leads to accumulation of β2 in the endoplasmic reticulum, along with impaired complex *N*-glycosylation, which is needed for its efficient surface delivery. We also show that these alterations to β2 affected to a certain extent Na_V_1.5 surface localization. Conversely, however, Na_V_1.5 had little or no influence on β2 trafficking, its localization to the surface, or homodimer formation. Altogether, our data link the architecture of the β2 domains to the establishment of its proper subcellular localization. These findings could provide valuable insights to gain a deeper comprehension of the elusive biology of β subunits in excitable cells, such as neurons and cardiomyocytes.

Many genetic disorders can bring about cardiac arrhythmias. Often, these are not associated with structural changes in the heart but can lead to sudden death. They are considered inherited channelopathies caused by pathogenic gene variants coding for ion channels that give rise to alterations in the generation or propagation of the action potential. In this regard, the altered functioning of voltage-gated sodium (Na_V_) channels can lead to cardiac channelopathies (1, 2).

Affected intracellular trafficking of ion channel components is a cause of channelopathies associated with inherited arrhythmias. One of the best-studied channelopathies is Brugada Syndrome, in which 20 to 25% of cases are due to a disease-associated variant in *SCN5A* (3); this gene encodes for Na_V_1.5, the pore-forming α subunit of the major cardiac Na_V_ channel (4, 5). The molecular mechanisms implicated are not clear but are essential to understanding arrhythmias and for the development of new therapeutic strategies in disease prevention and treatment. Among various proteins within the channel’s complex, Na_V_1.5 is associated with β subunits, which have long been believed to regulate the channel’s current density (6, 7, 8, 9).

The β subunit family consists of four genes: *SCN1B*, *SCN2B*, *SCN3B*, and *SCN4B.* Respectively, these encode β1–4; in addition, β1 has two alternative splice variants, *i.e.,* β1A and β1B. The β subunits are single-pass transmembrane proteins of a little more than 200 residues, whose extracellular immunoglobulin (Ig)-like domain forms an N-terminal loop to which adhesive properties have been attributed (10, 11, 12). Despite their implication in controlling the electrical activity needed for channel functioning in excitable cells, whether they play a more fundamental task has not yet been clarified. Nevertheless, recent studies emphasize the role of β subunits in modulating the channel’s biophysical properties (13), α subunit folding and localization (14, 15), and electrical conduction, more indirectly, through regulation of gene expression (16).

The topic of regulating trafficking and cell surface localization of α subunits is intriguing, particularly for β1 and β2, since the importance of both subunits has been reported, not only in initial studies in heterologous expression systems but also *in vivo* (17). Thus, cell surface expression of Na_V_ channels at the neuron axonal initial segment has been seen reduced in *Scn1b* null mice, thus lacking β1 (18). An implication in Na_V_ channel localization appears also particularly manifested for the β2 subunit. Accordingly, it has been reported, and supported by studies in *Scn2b* null mice, that β2 ensures α subunit delivery to the surface, and thus proper Na_V_ channel density, at the plasma membrane of neuronal cell bodies and myelinated axons (19), and also in intercalated discs of ventricular myocytes (20).

Taking into account this evidence, assembly and functioning of the Na_V_ channel appears to be influenced by accurate trafficking and localization of its associated β subunits. In consequence, in this work, we have continued our efforts to understand the regulation of β2 trafficking. To this end, in polarized Madin-Darby canine kidney (MDCK) cells, we have addressed the implication of the cytoplasmic tail, the extracellular loop, and the transmembrane domain (TMD) on proper folding, homodimer formation, trafficking, and cell surface localization of β2.

## Results

### Cell surface localization of β2 requires its cytoplasmic tail and the integrity of the extracellular loop

**Figure 1:**
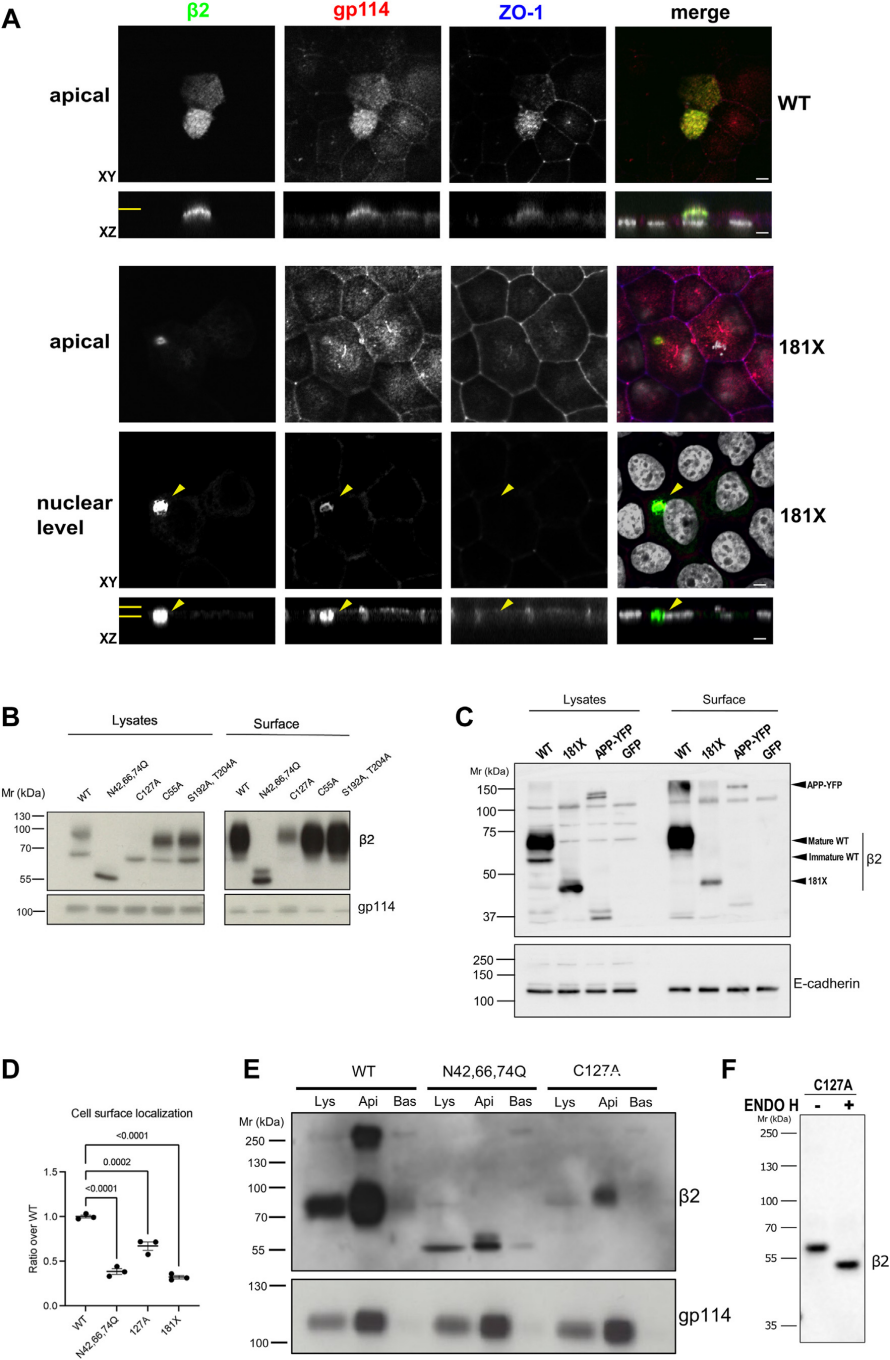
**Lack of the cytoplasmic tail or extracellular loop disruption impairs complex *N*-glycosylation of β2 needed for its efficient surface delivery.***A*, representative confocal XY sections of polarized cells taken at the apical plasma membrane, or deeper into the nuclear level (for 181X only), show strong overlap of transiently transfected β2-YFP (*green*) WT with apically located gp114 (*red*). On the contrary, the 181X mutant remains in dense intracellular aggregates (*arrowheads*). Normal pattern of the tight junction marker ZO-1 further confirms proper cell polarity (*blue*). *Horizontal yellow lines* in the corresponding *z*-axis reconstructions (XZ) mark the cell sections shown by XY. In merged images, nuclear staining by DAPI is in *grey*. Scale bars are 1 μm. *B* and *C*, representative Western blot after cell surface biotinylation showing plasma membrane levels of various β2 mutants compared to the WT in transiently transfected cells. *C*, as irrelevant controls, cells were transfected to express APP-YFP or simply GFP. Surface markers, gp114 (*B*) and E-cadherin (*C*), were blotted to normalize quantifications. *D*, band quantitation of mutants whose surface localization was visibly affected confirms reduction of the plasma membrane fraction in the C127A and 181X mutants, similarly to unglycosylated β2 (N42,66,74Q). Data are mean ± S.D, expressed as ratio over the WT. One-way ANOVA with Dunnett’s *post hoc* test revealed differences among means (*p* values comparing with the WT are shown). *E*, representative Western blot after cell surface biotinylation showing reduced, yet maintained, apical (Api) localization of β2 C127A, comparable to that of β2 N42,66,74Q; Bas indicates basolateral and Lys indicates lysate. Note that, once cells have achieved full confluence in Transwells (here, at 3 days from transfection), most of the C127A mutant is present in its mature form. *F*, representative western blots showing full cleavage (deglycosylation) by Endo H of β2 C127A, consisting mainly of immature *N*-glycans at 1 day post-transfection.

**Supplemental Figure S1:**
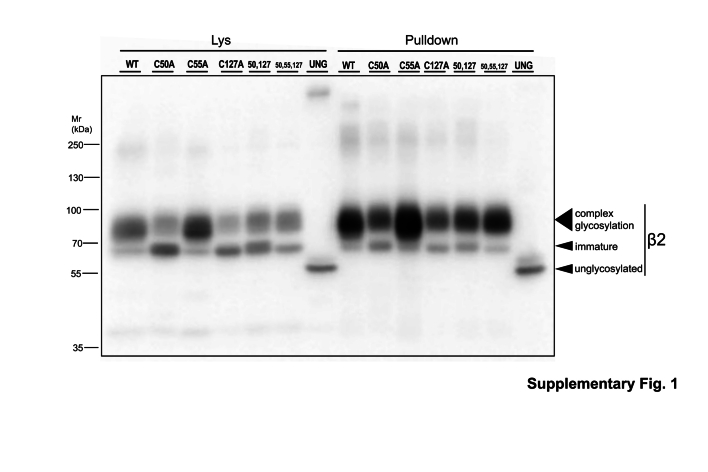


We have previously shown that removal of the C-terminal intracellular domain (ICD) of β2 makes the protein very mobile in the plane of the membrane; thus, while only 50 to 60% of β2 wild type (WT) can diffuse, β2 lacking its ICD, *i.e.*, truncated at residue 181 (181X), is completely mobile. As expected (21), a substantial portion of β2 181X appeared stuck intracellularly, in contrast to the WT (Fig. 1*A*), located at the apical surface in polarized MDCK cells, as also confirmed by cell surface biotinylation (Fig. 1, *C* and *D*). This agrees with our previous data since we had seen that a relatively small portion of the mutant localizes apically, remaining mostly in the endoplasmic reticulum (ER) (21). Interestingly, we found here a comparable behavior in β2 mutated to Ala in Cys-127 (C127A), thereby unable to form the major extracellular intramolecular disulfide bridge (Fig. 1, *B* and *D*), and therefore with the Ig-like loop disrupted. Still, as the unglycosylated β2, all surface C127A was at the apical domain (Fig. 1*E*). Like β2 181X (21), it was clearly defective in glycosylation, since Endo H cleaved most of its sugar portion, consisting of immature *N*-glycans generated in the ER (Fig. 1*F*); this may explain its impaired delivery to the cell surface. A comparable behavior was seen in β2 mutated to Ala in Cys-50 (C50A; Fig. S1). On the contrary, neither the C55A mutation, affecting the Cys residue shown to establish a disulfide bond with α subunits (22), nor abolishing residues at the ICD predicted to be phosphorylated (23), caused any effect (Fig. 1*B*).

**Supplemental Figure S2:**
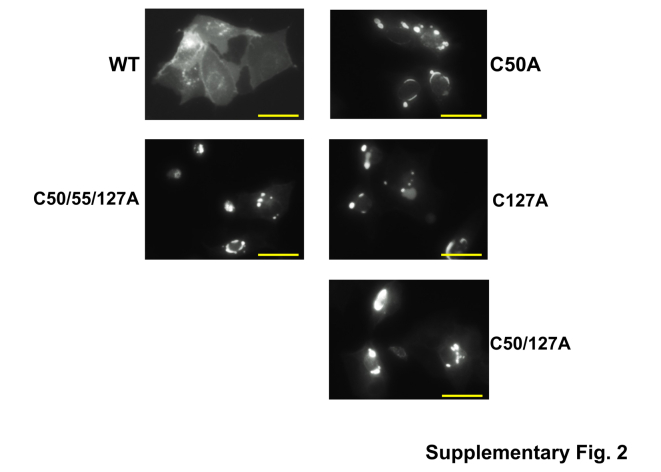


Interestingly, the triple Cys mutant, *i.e.*, with all three extracellular Cys residues mutated, often appeared visibly less affected than the C50A and C127A single or double mutants, which shared a substantial portion with immature glycosylation (Fig. S1). Moreover, in all these Cys mutants it was obvious that the proportionally minor fraction undergoing full (complex) glycosylation was the one reaching the cell surface more favorably (Fig. 1*B* and Fig. S1). In the C50A mutant, we cannot rule out that an intramolecular disulfide bridge could form between the neighboring Cys-55 and Cys-127, providing that Cys-55 remains sufficiently exposed on the molecule. However, all cells expressing β2 either with one, or both, of these extracellular Cys residues mutated, displayed comparably large intracellular aggregates, suggesting a major protein folding defect in all cases (Fig. S2).

These phenotypes are reminiscent of the behavior seen in unglycosylated β2, *i.e.,* mutated to Gln in the three Asn residues available for *N*-glycosylation (N42Q, N66Q, and N74Q), which also largely localized intracellularly (Fig. 1, *B*, *D*–*E* and Fig. S1), as we have previously reported (24). Therefore, the absence of the cytoplasmic tail or extracellular loop disruption prevented efficient delivery of β2 to the cell surface. Consequently, we hypothesized that the accumulation of these mutants in intracellular organelles, mostly in the ER, is responsible for their defective *N*-glycosylation, thereby impairing targeting to the cell surface, comparably as we found previously for fully unglycosylated β2 (24).

### Apical localization of β2 is influenced by its transmembrane domain

**Figure 2:**
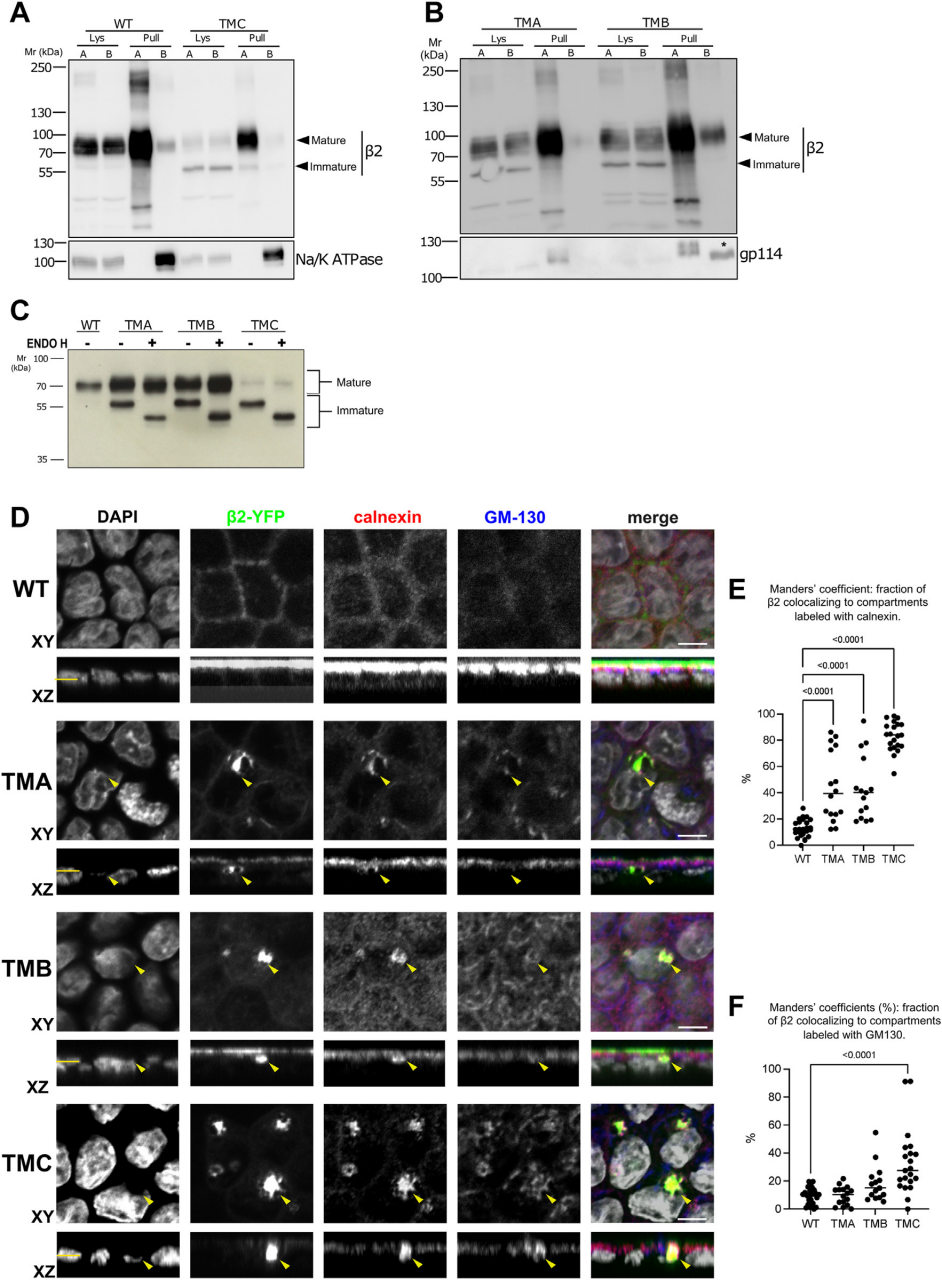
**Shortening of the β2 transmembrane domain affects glycosylation and leads to its accumulation in the endoplasmic reticulum.***A* and *B*, representative western blots after cell surface protein biotinylation of polarized cells stably expressing β2-YFP show apical localization also of TMD mutants. However, whereas their immature, still unprocessed, band is apparent in lysates, it does not reach the cell surface. Note that the TMC mutant expresses proportionally much lower levels of mature, fully glycosylated β2, than the WT, or even than the other mutants; this is the only form reaching efficiently the cell surface. Evidence of polarity is shown by proper surface localization of basolateral (B) Na/K-ATPase (*A*) or apical (A) gp114 (*B*) in pulldowns (Pull); Lys indicates lysate. Note that apparent traces of gp114 in the basal region (marked by an asterisk) come from a previous blot signal to Na/K-ATPase that could not be completely stripped off. *C*, representative western blot showing full cleavage (deglycosylation) by Endo H in all TMD mutants of the immature form, which is the main constituent of β2 TMC. *D*, representative confocal XY sections and corresponding *z*-axis reconstructions (XZ) of polarized cells stably expressing β2-YFP show, in contrast to the apical location of β2 WT, cytoplasmic accumulations of TMD mutants (*green*). These overlap visibly in the perinuclear region (*arrowheads*) with the ER marker calnexin (*red*), while not as much with the cis-Golgi marker GM130 (*blue*). In merged images, the nuclear staining by DAPI is in grey. Horizontal yellow lines in the corresponding *z*-axis reconstructions (XZ) mark the cell sections shown by XY. To emphasize the overlap of the β2 mutants with the markers tested, the XY sections displayed are those from the nuclear region. Scale bars are 5 μm. The dot plot showing Manders’ coefficients (expressed in %) indicates the fraction of β2 overlapping to compartments labeled with calnexin (*E*) or GM130 (*F*). Number of cells analyzed: n ≥ 15, from at least four different slides. One-way ANOVA with Tukey’s HSD *post hoc* test revealed differences among means (*p* values comparing with the WT are shown); the mean is displayed as a horizontal bar.

To dissect further the regions or domains on β2 implicated in its delivery to the cell surface, and particularly in its polarized targeting, we next investigated the role of the TMD. To analyze the effect of its length, we engineered three mutants with a shortened TMD, each by gradually taking out five residues along the segment. That is, mutants TMA, TMB, and TMC lack five residues, respectively, from the outer leaflet of the plasma membrane toward the cytoplasmic leaflet. Despite the deletion, all still presented mostly unaltered apical localization, undistinguishable from that of β2 WT (Fig. 2, *A* and *B*). Similarity with the WT was indeed obvious for TMA and TMB (Fig. 2*B*). Yet, all mutants shared a visible fraction of immature, still unprocessed, β2 most of which could not progress beyond the ER, as confirmed by Endo H treatment (Fig. 2*C*). As previously seen (24), such unprocessed β2 was virtually absent at the plasma membrane. This was clearly manifested in the TMC mutant (Fig. 2*A*). Here, the fraction that actually reached the apical surface was essentially the fully glycosylated β2 (mature), albeit markedly underrepresented in whole cell lysates, especially in comparison with the WT. Quantitation of these blots led to the estimation that the relative amount of β2 reaching the apical surface (over total cellular β2) is about 50% higher in the WT than in the TMC mutant. These results suggest that the stretch missing in the TMD closest to the cytoplasm plays a more prominent role in cell surface delivery and thus in the apical localization of β2. This also confirms that β2 must be completely glycosylated to reach the surface efficiently. In this regard, a glycosylation defect was indeed more apparent in β2 TMC than in the other two mutants (Fig. 2, *A*–*C*).

### Shortening of the transmembrane domain leads to β2 accumulation in the endoplasmic reticulum

Previous work has shown that shortening of a protein’s TMD leads to intracellular localization, such as in the ER or the Golgi apparatus (25). We thus addressed colocalization with organelle markers by immunofluorescence and confocal microscopy to understand further the intracellular retention of the TMD mutants. Indeed, all displayed considerable overlap with the ER marker calnexin, which was not as obvious when co-staining with the Golgi matrix protein of 130 kDa (GM130), a typical cis-Golgi marker. Moreover, retention was more manifested in the TMC mutant, indicating its greater impairment in apical targeting (Fig. 2*D*). To estimate the degree of overlap in cells, we calculated the Manders’ coefficient along the *z*-axis. In the TMC mutant, the coefficient often reached 0.8 for calnexin at nuclear sections, or right above the nucleus, with very little presence at the cell surface, indicating that at least 80% was present in ER compartments (Fig. 2*E*). Overlap with GM130 was overall not as marked, yet still apparent for this particular mutant (Fig. 2*F*), while limited progress beyond the Golgi apparatus is consistent with the digestion achieved with Endo H (Fig. 2*C*).

### Cys-55 serves for β2 oligomerization although monomeric β2 can still localize to the cell surface

**Figure 3:**
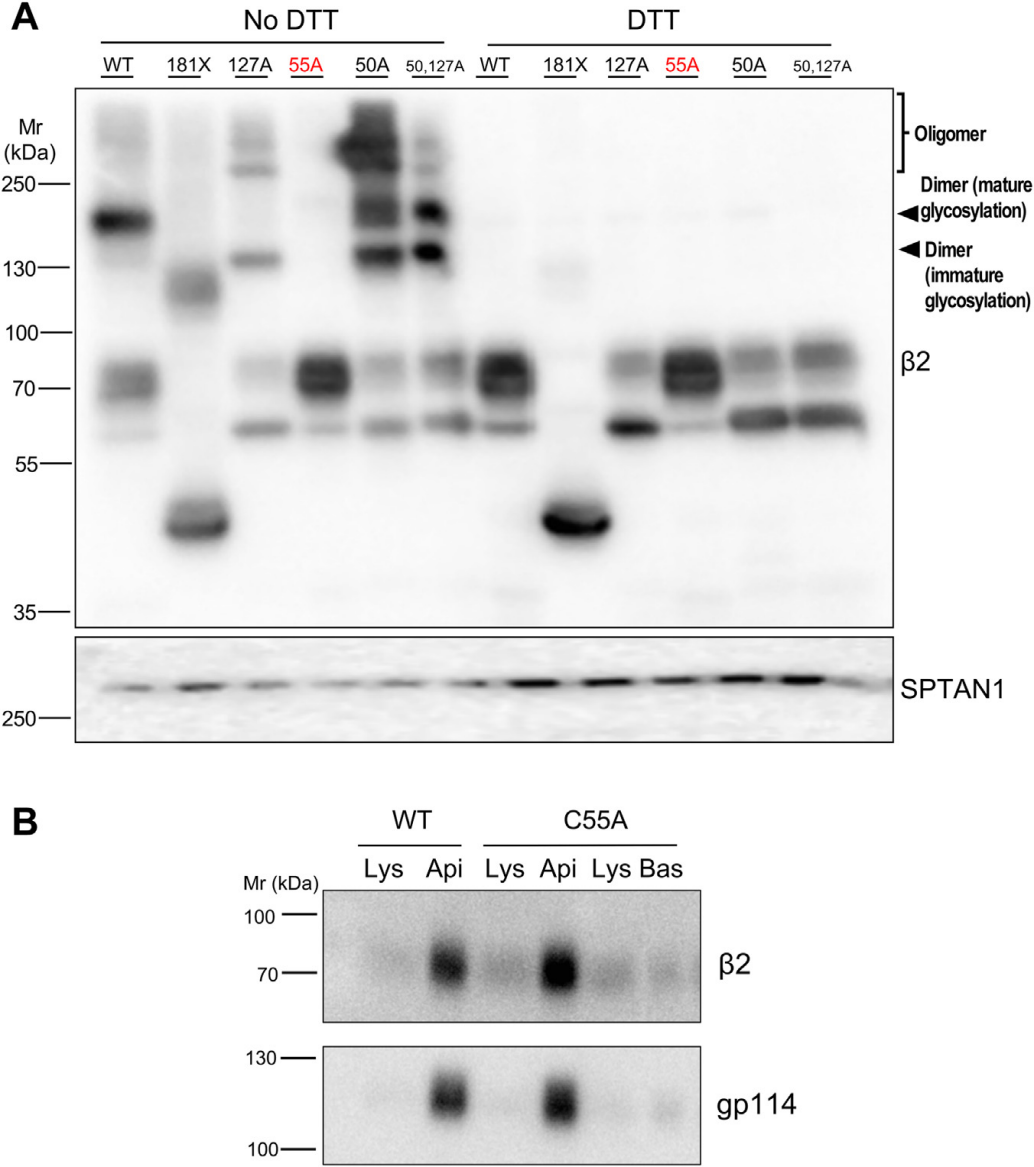
**β2 oligomerizes through Cys-55.***A*, representative western blots of lysates from cells transiently expressing WT or mutant β2-YFP, analyzed in reducing (DTT) or non-reducing conditions (No DTT), show that the C55A (in red fonts) is the only mutant unable to dimerize. Oligomerization is manifested by the presence of high molecular weight bands displaying a pattern coincident with that of dimers fully glycosylated and others with immature glycosylation. High-order oligomers, likely tetramers, are also observed in C50A and C127A mutants. In all Cys mutants shown here, this residue was replaced by Ala (A). Upper and lower bands seen in reducing conditions are, respectively, fully glycosylated and immature monomeric β2. The blot to α-spectrin-1 (SPTAN1) serves as a loading control. *B*, representative western blots after cell surface protein biotinylation of transiently transfected cells show apical localization (Api) of β2 C55A. Evidence of polarity is confirmed by proper apical localization of gp114; Bas indicates basolateral and Lys indicates lysate.

At the β2 extracellular domain, Cys-55 establishes a disulfide bond with α subunits (22). As a prerequisite for apical targeting, it is possible that *N*-glycosylation would participate in the stabilization of dimerized β2, providing that it could covalently interact with itself through a disulfide bond. Therefore, we next addressed β2 dimerization and whether such property was affected in the mutants studied here. To this end, cell lysates were analyzed by Western blot under non-reducing conditions. C50A and C127A mutants displayed a similar pattern of high-order oligomers, comparably reduced in the double mutant and absent in the WT. It is possible that, in forms lacking an intramolecular bridge, Cys-55 remains more exposed, thus favoring the assembly of perhaps four subunits, taking into account the observed apparent molecular weight. As expected (22), C55A was conclusively the only form unable to dimerize (Fig. 3*A*). Nevertheless, this mutant had no trouble reaching the plasma membrane, locating effectively at the apical surface, and thus behaving identically as the WT (Fig. 3*B*). Interestingly, we observed that β2 181X can also dimerize (Fig. 3*A*), despite undergoing complete Endo H cleavage, consistent with its ER localization (21).

### Monomeric, non-palmitoylated β2 localizes to the apical domain

**Figure 4:**
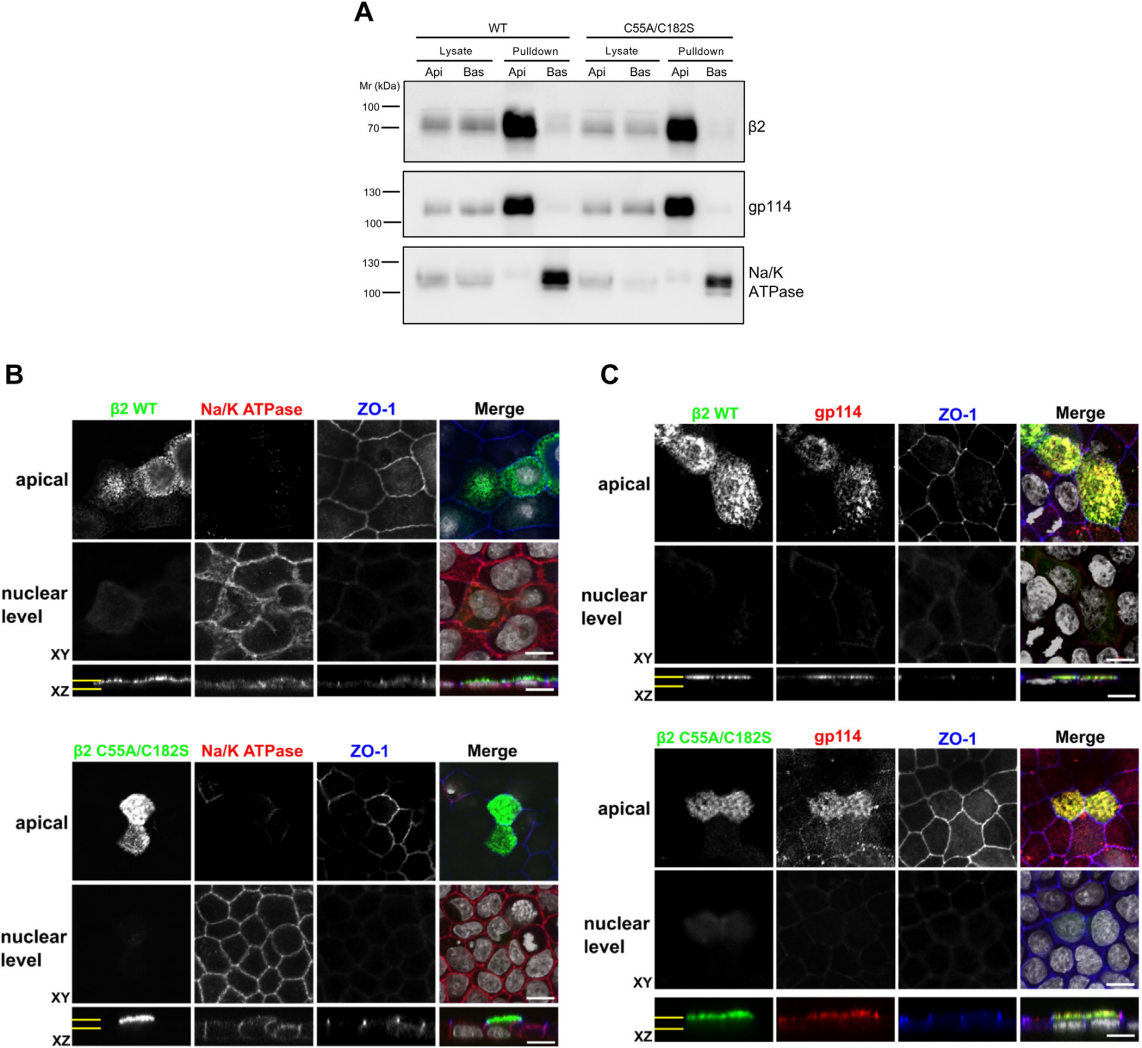
**Mutation on both Cys-55 and Cys-182 does not affect β2 apical localization.***A*, representative Western blots after cell surface protein biotinylation of polarized cells transiently expressing β2-YFP show apical localization of the mutant C55A/C182S in a manner indistinguishable from the WT. Markers gp114 and Na/K-ATPase remain at their apical (Api) and basolateral (Bas) domains in pulldowns, respectively, confirming proper cell polarity. *B* and *C*, representative confocal XY sections and corresponding *z*-axis reconstructions (XZ) of polarized cells transiently expressing β2-YFP show that both WT and the C55A/C182S mutant (green) are exclusively located at the apical section, merging with gp114 (*C*), but not with Na/K-ATPase (*B*), the latter, well visible at the nuclear level (both surface markers are in *red*). The normal pattern of the tight junction marker ZO-1 further confirms proper cell polarity (*blue*). The two parallel yellow lines in XZ mark the cell sections shown by XY, either apical or nuclear. In merged images, the nuclear staining by DAPI is in grey. Scale bars are 10 μm.

So far, our data show that cytoplasmic tail and extracellular loop are required for β2 surface delivery, with the TMD also playing an important part. Yet, dimerization does not appear to be a requirement. We previously found that palmitoylation of Cys-182—located at the boundary with the membrane bilayer—influences β2 mobility in the plane of the membrane. Our data suggested that palmitoylation might contribute—within a liquid-ordered, cholesterol-rich environment—to establishing the polarized distribution of β2 (21). Consequently, we hypothesized here that monomeric and non-palmitoylated β2, *i.e.,* the double mutant C55A/C182S, could be defective in proper surface localization. Yet, we found that it did not display any measurable defect in this regard, localizing at the apical region in a manner indistinguishable from β2 WT, as seen by cell surface protein biotinylation (Fig. 4*A*) and confocal microscopy (Fig. 4, *B* and *C*).

### Shortening of the β2 transmembrane domain hampers dimerization

**Figure 5:**
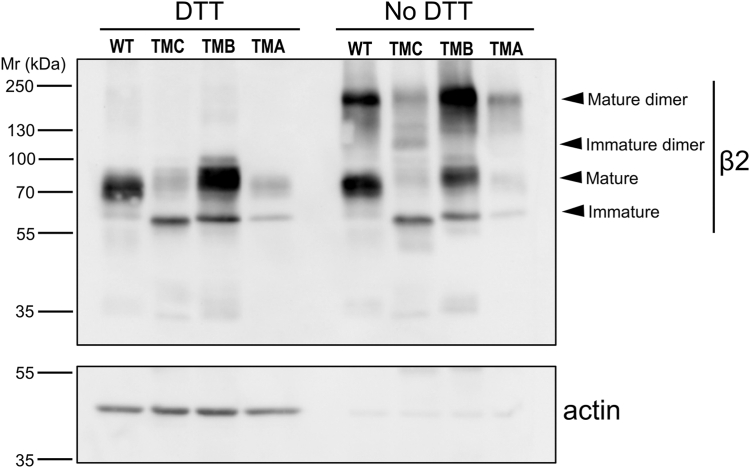
**The TMD mutant TMC displays a dimerization defect.** Representative western blots of lysates from cells stably expressing β2-YFP, analyzed in reducing (DTT) or non-reducing conditions (No DTT), show that the WT, as well as the TMA and TMB mutants, display a considerable proportion of β2 as a mature (fully glycosylated) dimer. On the contrary, the TMC mutant exhibits a proportionally smaller fraction in comparison with levels of its immature (ER-glycosylated) monomeric form; the bands at around 120 kDa and above likely correspond to a minor portion of dimerized TMC mutant displaying different degrees of glycosylation (band pattern marked by arrowheads). Note that the mutant TMA, while expressed at comparatively lower levels than the other β2 forms, displays a considerable portion of the mature dimer. Although inefficiently solubilized in the absence of DTT, actin serves as a loading control.

Following our hypothesis on the key implication of *N*-glycosylation and TMD length in correct surface targeting of β2, we next addressed their possible connection in the ability to dimerize. As above, lysates were processed, in parallel, also in non-reducing conditions. The β2 WT, along with the TMA and TMB mutants—*i.e.*, those with minor defects on β2 maturation (see Fig. 2*B*)—showed proportionally abundant high molecular weight bands in non-reducing conditions, likely corresponding to fully glycosylated dimers. Instead, comparatively scant and rather spread bands were found in the TMC mutant, consistent with these being relatively scarce forms of the dimerized mutant, including a minor portion with complete glycosylation (Fig. 5). Therefore, the TMC mutant, displaying a proportionally important immature form, was somewhat defective in dimerization. Yet, while these data might be consistent with the idea that complex glycosylation is required for β2 to dimerize, tail-minus (181X) and Ig-loop disrupted mutants, while all clearly defective in complex glycosylation, can still form dimers (see Figs. 1, *B* and *F*, 3*A* and S1). Therefore, β2 dimerization takes place before and/or independently from mature glycosylation, although protein-folding defects, such as the result of TMD shortening, can affect these processes.

### Affected Na_V_1.5 localization to the plasma membrane with mutated β2

**Figure 6:**
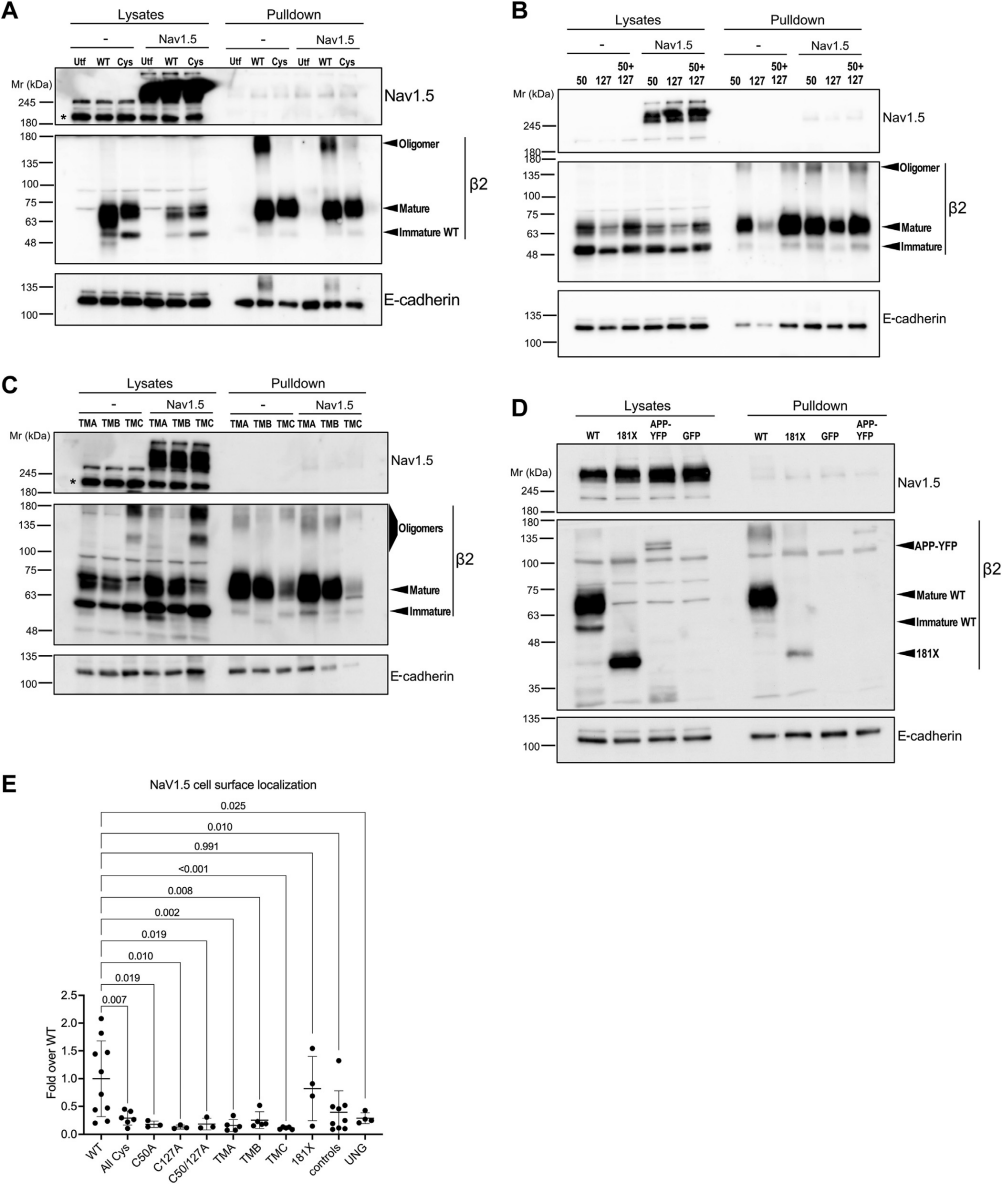
**Effects on Na**_**V**_**1.5 localization to the plasma membrane in the presence of mutated β2.** Representative Western blots after cell surface protein biotinylation of parental cells (*A*–*C*: −), or stably expressing Na_V_1.5-YFP (*A*–*D*: Na_V_1.5), and transiently expressing WT or mutant β2-YFP, reveal slight reductions in plasma membrane levels of Na_V_1.5 when β2 was mutated; this effect could be estimated, confirming a decrease by all mutants except for β2 181X (*E*, *p* values are shown). Conversely, glycosylation pattern and surface distribution of the various β2 forms (marked by *arrowheads*) appears unaltered; dimers are referred on the image as oligomers. As irrelevant controls, cells were transfected to express APP-YFP, GFP (*D*), or left untransfected (*A*: Utf). A blot to E-cadherin is included as loading control to adjust for fluctuation in solubilized material. Non-specific band(s) above the 180 kDa marker in lanes of parental cells are labeled with an asterisk. While the same amount of plasmid DNA was used for all transfections, band intensity may vary depending on the stability of each mutated β2; note for instance that the C127A mutant (*B*, 127) tends to be poorly expressed, but behaves comparably to β2 C50A (50) and C50A/C127A (50 + 127). Cys indicates the C50A/C55A/C127A triple mutant and UNG unglycosylated β2.

The data shown underline the important implication of cytoplasmic tail, extracellular loop, and TMD on proper β2 folding and export to the cell surface in polarized MDCK cells. Yet, we have performed all experiments without considering the connection of β2 with Na_V_1.5, *i.e.*, the pore-forming α subunit of the major cardiac Na_V_ channel, whose gene variants account for arrhythmia-associated pathologies (3). Given that such association may modulate α subunit trafficking, as reported even in mouse models (19, 20), we asked whether the alterations studied here on β2 affect Na_V_1.5 localization to the cell surface. To this end, we took advantage of a cell line stably expressing YFP-tagged Na_V_1.5 (26). In these cells, we transiently expressed each of the β2 forms analyzed here. Consistent with our previous data (24), we found, by cell surface biotinylation, a measurable increase, albeit modest, in plasma membrane Na_V_1.5 in the presence of β2 WT. However, this was not seen with most of the mutants. This applies to those with the extracellular loop disrupted or the TMD shortened, while cytoplasmic tail deletion appeared to have no effect (Fig. 6*E*). Conversely, the pattern of the various β2 mutants remained unaffected in the presence of Na_V_1.5, namely, their degree of glycosylation, localizing to the surface to a similar extent (Fig. 6; see also Fig. 1*C*, displaying the 181X mutant alone).

**Figure 7:**
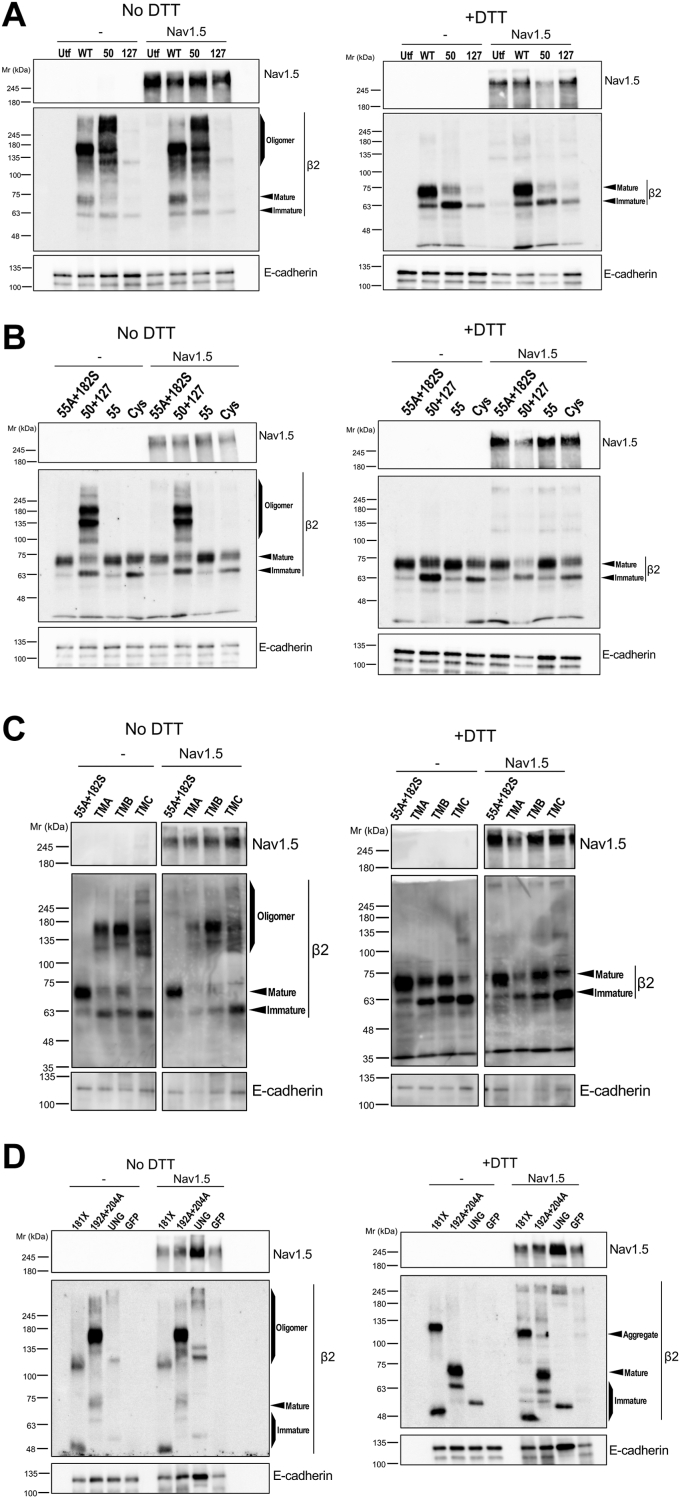
**All β2 variants display a comparable homodimerization pattern in the presence of Na**_**V**_**1.5.** Representative western blots of lysates from parental cells (−), or stably expressing Na_V_1.5-YFP (Na_V_1.5), and transiently expressing WT or mutant β2-YFP, were analyzed in reducing (+DTT) or non-reducing conditions (No DTT), showing a comparable oligomerization pattern of the various β2 forms with and without Na_V_1.5. The band pattern is marked by arrowheads, with oligomers also referring to higher-order forms, likely tetramers, such as the band above the 245 kDa marker manifested in the C50A mutant (*A*, 50). As irrelevant controls, cells were transfected to express GFP (*D*), or left untransfected (*A*: Utf). A blot to E-cadherin is included as loading control to adjust for fluctuation in solubilized material. While the same amount of plasmid DNA was used for all transfections, band intensity may vary depending on the stability of each mutated β2; note for instance that the C127A mutant (*A*, 127) consistently tends to be poorly expressed, yet displaying a pattern comparable to that of β2 C50A. Note that, in reducing conditions, a pale pattern of nonspecific bands can be seen above β2-YFP in the lanes with Na_V_1.5; that was due to low molecular weight bands showing with the anti-GFP antibody. Note also that a high molecular weight form of the 181X mutant is marked as aggregate in reducing conditions (D: +DTT). Cys indicates the C50A/C55A/C127A triple mutant; 55A + 182S, C55A/C182S; 50 + 127, C50A/C127A; 55, C55A; 192A + 204A, S192A/T204A; and UNG, unglycosylated β2.

Despite the lack of an effect on surface delivery, we reckoned that it would still be possible that β2 self-assembly was affected by Na_V_1.5, primarily, taking into account the previously reported α/β2 covalent interaction (22). However, all β2 mutants studied here displayed a comparable dimerization pattern irrespective of Na_V_1.5 coexpression (Fig. 7). Taken together, these data in MDCK cells suggest that the interaction of β2 with Na_V_1.5 is complex and occurs differently than in other Na_V_ channel isoforms, consistent with current structural evidence (27).

### The tail-minus β2 mutant is not endocytosed

The accurate polarized location of β2 may be determined by well-regulated dynamics and stability at such a location. The seemingly simple architecture of β subunits would favor a plasma membrane localization, although that does not seem to apply to all β subunits, particularly β1 (16, 26) and β3 (Fig. S3), both of which remain mostly intracellular in immortalized cells, likely due to differences with β2 in their sorting motifs. As for β2, once at the surface, we have observed that it displays slow dynamics within the membrane and does not get endocytosed (21). Here, we hypothesized that the lack of endocytosis is caused by anchoring to the sub-membrane cytoskeleton through its cytoplasmic tail.

**Figure 8:**
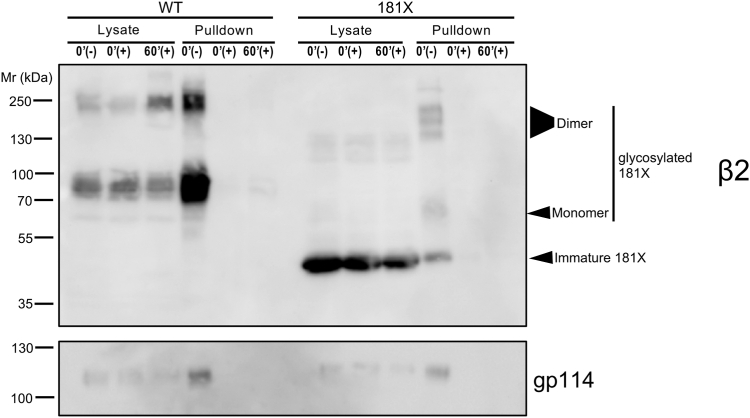
**The tail-minus mutant does not undergo endocytosis.** Representative western blots of polarized cells stably expressing β2-YFP after cell surface biotinylation show the absence of WT and the 181X mutant in pulldowns after 60 min (60′) from glutathione treatment (+), denoting lack of apical endocytosis. Pulldowns at t = 0 min (0′), before glutathione treatment (−), indicate total apically biotinylated β2. As a control, note absence of biotinylated protein in pulldowns of cells treated with glutathione (+) immediately after labeling (0′). As expected, apical gp114 does not get endocytosed either.

As β2 181X was poorly expressed, we added a (Gly-Gly-Ser-Gly)_2_ linker between residue 181 and the YFP tag (21). Moreover, we used stable cell lines for endocytosis assays to ensure sufficient expression levels. Thus, we biotinylated apical membrane proteins and examined their endocytosis upon glutathione cleavage of the biotin reagent; endocytosis leaves intracellular moieties protected. A visible portion of the 181X mutant reached the cell surface as a glycosylated monomer or dimer (Fig. 8, pulldown at time zero). As expected, the label from biotinylated β2 WT, effectively gone after glutathione cleavage at time zero, remained virtually undetected in pulldowns during the chase period. Similarly, the 181X mutant also remained undetected; this indicates that, even when lacking its ICD, β2 still displays very little or no endocytosis in MDCK cells (Fig. 8). Because of the completely mobile nature of β2 181X (21), these data suggest that the limited β2 dynamics in the plane of the membrane is likely unrelated to its rate of internalization.

## Discussion

**Figure 9:**
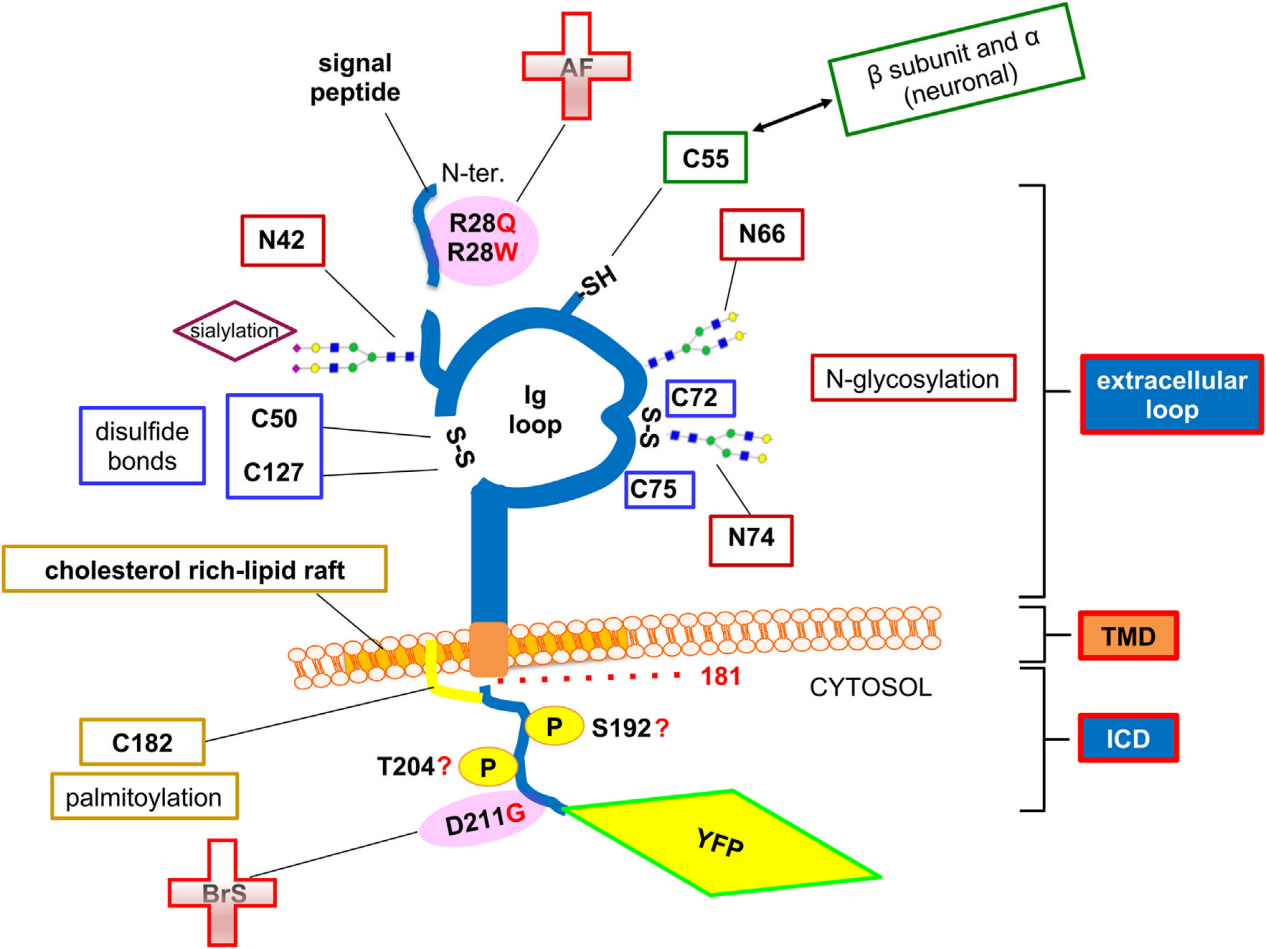
**Cytoplasmic tail, extracellular loop, and transmembrane domain all contribute to proper β2 trafficking and localization.** Cartoon highlighting the portions of YFP-tagged β2 determinant for its proper trafficking to the cell surface and polarized localization. Cytoplasmic tail, shown as the ICD; extracellular loop, where the major intramolecular disulfide bond forms the Ig loop; and TMD, with a tendency to locate in cholesterol-rich raft domains, are highlighted as important regions for β2 trafficking and localization. Mutations found for cardiac-associated arrhythmias, *i.e.*, atrial fibrillation (AF), on Arg-28 (56), and Brugada Syndrome (BrS), on Asp-211 (57), are also shown. See additional details in the text.

In this work, we analyzed portions in the architecture of the Na_V_ channel β2 subunit that we predicted would ensure its proper trafficking and polarized surface localization. We provide evidence that the presence of the ICD, TMD length, and integrity of the extracellular loop are three major components implicated (Fig. 9).

β2 unambiguously localizes to the cell surface in many cell types studied (22, 26, 28, 29), including in myocytes from mouse ventricles (30) and human atria (31), although surprisingly not in neonatal rat cardiomyocytes (31). We have shown that β2 localizes in a polarized fashion if exogenously expressed in polarized MDCK cells. Specifically, it is delivered exclusively to the apical surface (26), although it is not entirely clear how. This selective distribution could be important in excitable cells, such as for Na_V_ channel localization to nodes of Ranvier and the axon initial segment in neurons. Moreover, β subunits mediate cell adhesion, which appears critical for neuron proliferation and migration (32). Intriguingly, a hypothetical polarized distribution of β subunits in cardiomyocytes has not been studied in detail. Nonetheless, in isolated mouse ventricular myocytes, both β1 and β3 were detected in transverse tubules, whereas β2 and β4 were in intercalated disks, perhaps along with a major pool of Na_V_1.5 (30). Indeed, we have proposed that polarized delivery of β2 could occur, upon *N*-glycosylation of its luminal domain, through recognition by sugar-binding galectins (29). On the other hand, an association of its TMD with lipid rafts appears to have a role in β2 dynamics and thus possibly in membrane subdomain distribution. In this regard, we have provided evidence that it is acylated at Cys-182, a residue located at the boundary with the membrane bilayer, whose mutation affected β2 association with detergent-resistant membranes (21), which are enriched in lipid rafts (33).

Here, when addressing the role of the TMD on β2 localization to the cell surface, we were also inspired by previous findings showing association with lipid raft domains of β subunits (34) and the Na_V_1.5-based channel complex (35). In fact, the length of a protein’s TMD, equivalent to a stretch of hydrophobic amino acids, appears important for proper partitioning within membrane subdomains (36). TMDs of plasma membrane proteins tend to be longer than in ER- and Golgi-resident proteins. In the models proposed, a thicker surface domain, enriched in ordered lipids, also appears more suitable to accommodate proteins with longer TMDs (36). Here, we hypothesized that shortening the TMD would cause β2 to remain in the cytoplasm. Consequently, a visible portion of β2 was indeed retained intracellularly, although the remaining protein was still delivered apically. Nevertheless, shortening of the TMD not only affected dimerization but, more importantly, glycosylation, suggesting poor progress along the ER-to-cell surface pathway. Interestingly, this was more dramatic in the mutant with the deletion closer to the cytoplasmic leaflet, referred to here as TMC. Perhaps, the defect is associated with the degree of hydrophobicity of the residual TMD. A weakly hydrophobic stretch would render a protein more difficult to anchor to the lipid bilayer, possibly affecting already its initial folding at the ER. In consequence, this could reduce the efficiency of delivery to the plasma membrane. However, adding up the hydropathy index (or hydrophobicity score) of the residues along each TMD stretch (37), we have found their score to be 47.4 for the TMA mutant, 54.5 for TMB, and 48.0 for TMC. Thus, the latter appears, respectively, almost identical or lower than the TMA and TMB mutants, both much less affected. At any rate, there can be additional elements involved. Thus, defective *S*-palmitoylation on Cys-182 may also occur, in the TMC mutant, due to proximity to the stretch of residues deleted, *i.e.*, from 176 to 180 (21); in this context, this could affect its export to the cell surface. Overall, we favor the idea that defective folding at the ER—in the current situation, because of TMD shortening—slows down subsequent β2 export to the cell surface.

It is worth underlining the very striking phenotype of ER retention seen in unglycosylated β2 (24). Such a defect is comparable to what we have found in the current work in β2 lacking the ICD or with a disrupted extracellular Ig loop. We reckoned that the role of the extracellular loop in the trafficking and localization of β2 could be addressed by mutating the Cys residues participating in intramolecular disulfide bridge crosslinking, which would thereby eliminate the loop. This has been previously investigated in the disease-causing variant of β1, C121W—mutated at the equivalent site of β2 Cys-127—whose alteration would also lead to disruption of the loop architecture. Interestingly, however, while C121W prevented β1 from localizing to the axon initial segment, localization of the Na_V_ α subunit was not affected (38). Here, we also attempted to investigate the link between β2 and Na_V_1.5, due to the major relevance of Na_V_1.5 in human heart function (5). Moreover, as in neurons (19), there is evidence that β2 chaperones the α subunit to the cardiomyocyte sarcolemma (20).

In agreement with these studies—including our own previous work in MDCK cells (24)—we have measured here an effect on Na_V_1.5 plasma membrane localization in the presence of β2 WT. Such effect appeared diminished when coexpressed with β2 either with the extracellular loop disrupted or with the TMD shortened, albeit remained unchanged if the ICD was deleted. While such a reduction of Na_V_1.5 surface localization was quantifiable, it was not striking, at least in our system. This could certainly derive from the limitation of MDCK cells – which we must highlight here—to study the biology shared by α/β subunits. Nonetheless, regarding Na_V_1.5 in particular, structural data have led to the proposal that it cannot form the same regulatory interface with β subunits as other α subunits do (27). As it has been suggested, β subunits—including β2—likely interact with Na_V_1.5 in a different way than in other Na_V_ channels (12). Somehow consistent with their singular connection, the cell surface localization and dimerization pattern of the various forms of β2 studied here remained unchanged in the presence of Na_V_1.5.

Regarding dimerization, in β4, for instance, it takes place through Cys-58 (39). Thus, one would expect that β subunits could form homodimers or even oligomers. In fact, it has been proposed that β3 trimeric assembly induces high-order clustering of the Na_V_ channel (40). Moreover, both *cis*- and *trans*-homophilic and heterophilic interactions between β subunits mediate their adhesion and mechanosensitivity properties (12). We show here that β2 dimerization takes place through Cys-55; this was expected since this site mediates disulfide bond formation with neuronal α subunits (22).

Interestingly, except for C55A, all mutants on extracellular Cys residues turned out defective in undergoing complex glycosylation, which uncovers their defect in progressing beyond the ER. It is possible that aberrant folding is the sole culprit here. Alternatively, lack of glycosylation by itself would prevent interaction with *e.g.* sugar-binding lectins, which could assist β2 to the cell surface and, specifically, to the apical domain. This matter, deeply studied in the past, remains an open question (41, 42, 43).

The contribution of the ICD in regulating β2 trafficking has also remained certainly unexplored. In this regard, we have speculated that its phosphorylation—for example, by casein kinase II—of cytosolic Ser-192 and Thr-204 (23) could mediate interactions with adaptors implicated in trafficking. Specifically, it could influence the recruitment of ankyrin G (29), as shown for β1, in which Tyr phosphorylation influences its localization in cardiac myocytes (44). As for β1, the Y200E mutation (Tyr changed to Glu), which mimics phosphorylation, abolishes ankyrin G recruitment, likely affecting Na_V_ channel localization to nodes of Ranvier (45). Thus, an interesting hypothesis is that aberrant phosphorylation of β2 would render this subunit to behave as the tail-minus mutant, abolishing interactions with cytosolic adaptors and becoming fully mobile in the plane of the membrane.

Conceptually, we have envisioned that the β2 cytoplasmic tail should contribute, leastwise, to retain the protein at a certain location. By anchoring β2 to the submembrane actin cytoskeleton, thereby restraining movement in the plane of the membrane, it could certainly influence its diffusion in the plane of the membrane (21). Here we have addressed whether a lack of the ICD would trigger β2 endocytosis. By completely removing its ICD, β2 was barely expressed. However, the expression did improve by adding a linker, which, by itself, is about one-fourth the length of the actual tail. To our surprise, the tail-minus mutant behaved identically to the WT, with no signs of internalization, at the least, of the comparatively reduced portion of this truncated form that can reach the plasma membrane. Yet, we conclude that β2 181X, similar to the unglycosylated mutant (24), must have reached the cell surface by bypassing the Golgi compartment, thus being limited to undergo immature glycosylation in the ER. Supported by evidence from electrophoreses performed in non-reducing conditions, limited glycosylation perhaps allows the mutant to self-interact. Therefore, it is possible that its oligomerization prevents internalization from the cell surface, which perhaps occurs in some analogous way also in β2 WT. As for the lack of effect observed by β2 181X in reducing plasma membrane Na_V_1.5, we hypothesize that it retains protein regions that may still allow some degree of interaction with Na_V_1.5 that the other mutants lack. This suggests that correct folding of the extracellular domain (mostly, due to its posttranslational modifications) and/or an intact TMD, and likely the resulting subcellular localization, appear determinant in establishing this link, likely weak and complex, with Na_V_1.5.

It is intriguing that the C55A monomeric mutant had no difficulties reaching the cell surface or localizing properly to the apical domain. Thus, we constructed a double mutant, so that, on top of being unable to dimerize, it could not undergo palmitoylation, therefore being defective to partition into detergent-resistant membranes (21). Still, we have found here that the β2 C55A/C182S double mutant localizes properly to the apical surface, not showing apparent differences with the WT.

Regarding the C50A/C55A/C127A triple mutant, it localized to the cell surface in a manner nearly comparable to the WT. A possible explanation is that a substantial portion manages to become fully glycosylated simply perhaps because the three modifiable Asn residues are left more exposed in the fully unfolded extracellular domain. Apart from the presence of a short disulfide bridge between C72 and C75—a unique feature of β2 among β subunits (46) —the Ig loop should be completely abolished upon elimination of these three Cys residues. As the C127A mutant, both the single C50A mutant and the C50A/C127A double mutant had on the contrary a clear glycosylation defect. On the other hand, even though the Ig loop architectural integrity is—at least intuitively—abolished by mutating C50 and/or C127, in the C50A mutant, because of its proximity to Cys-55, a disulfide bridge might be hypothetically formed between Cys-55 and Cys-127. Thus, we cannot rule out this possibility, providing that Cys-55 remains sufficiently exposed to the molecule. In principle, that would reestablish the Ig loop, at least partially. However, mutants C50A and C127A appeared indistinguishable from each other. In fact, we observed that cells expressing at least one extracellular Cys residue mutated, except for C55A, display comparably large intracellular aggregates. On the other hand, their dimerization was not affected, and the same applied to the C50A/C127A double mutant, perhaps simply because Cys-55 becomes more available for dimerization in the unfolded β2. Overall, we conclude that cell surface delivery, in particular to the apical domain, appears generally affected whenever β2 cannot achieve mature glycosylation.

In summary, in this work we present evidence that different regions of β2 contribute to its proper folding and ensure posttranslational modifications, particularly glycosylation, which appears required for its effective polarized surface localization. Thus, ICD, extracellular loop integrity, and TMD are all implicated to a considerable degree. As an important limitation of our study, we should bear in mind that the mechanisms observed here in MDCK cells may differ substantially and therefore not fully reflect what actually takes place in differentiated cardiomyocytes, or other excitable cells, whose complexity could not be reproduced in our study.

## Experimental procedures

### Plasmid vectors, cDNA cloning, and site-directed mutagenesis

The vector containing *SCN2B*-*yfp*, to express human β2 with YFP fused to its cytoplasmic C-terminus, has been previously described (26). Vectors containing *SCN1B*-*cfp*, to express rat β1 (28), and *SCN3B*-*gfp*, to express rat β3 (47), with the fluorescent tag fused to their cytoplasmic C-terminus, and the vector to express C-term YFP-tagged human amyloid precursor protein (APP) (48), have also been described.

Following the manufacturer’s instructions, the QuikChange Lightning Site-Directed Mutagenesis Kit (Agilent Technologies, Inc., Santa Clara, CA, US) was used to carry out the mutations of the current study. Human *SCN2B* (designated in the Consensus Coding Sequence database—CCDS—with ID 8390.1) was used as a template. Complementary primer pairs for PCR were designed with the QuikChange Primer Design Program (Agilent) and synthesized by Metabion International AG (Steinkirchen, Bavaria, Germany).

To generate the extracellular Cys mutants, Cys-50, Cys-55, and/or Cys-127 were changed to Ala. We created combinations of double and triple mutants. Primer sequences were as follows (mutated bases are marked in bold and underlined): for Cys-50, 5′- CGCCCGCCTGCCC**GCC**ACCTTCAACTCCT -3′ (sense) and 5′- AGGAGTTGAAGGT**GGC**GGGCAGGCGGGCG -3′ (antisense); for Cys-55, 5′- CCTGCACCTTCAACTCC**GCC**TACACAGTGAACCACA -3′ (sense) and 5′- TGTGGTTCACTGTGTA**GGC**GGAGTTGAAGGTGCAGG -3′ (antisense); and for Cys-127, 5′- GATGAGGGGATTTACAAC**GCC**TACATCATGAACCCCCC -3′ (sense) and 5′- GGGGGGTTCATGATGTA**GGC**GTTGTAAATCCCCTCATC -3′ (antisense). The mutant in which Cys-182 was changed to Ser has been previously generated (21).

To make β2 doubly mutated on the two residues predicted to be phosphorylated (23), both Ser-192 and Thr-204 were mutated to Ala. Primer sequences were as follows (mutated bases are marked in bold and underlined): for Ser-192, 5′- AAAAAAAGAGCAGAAGCTG**GCC**ACAGATGACCTGAAGACC -3′ (sense) and 5′- GGTCTTCAGGTCATCTGT**GGC**CAGCTTCTGCTCTTTTTTT -3′ (antisense); and for Thr-204, 5′- GGAGGAGGGCAAG**GCG**GACGGTGAAGG -3′ (sense) and CCTTCACCGTC**CGC**CTTGCCCTCCTCC -3′ (antisense).

To shorten the TMD, to generate each of the three mutants with five residues deleted within their TMDs, the same Mutagenesis Kit was employed. Forward and reverse primers were designed to flank each of the three regions to be deleted. From the outer leaflet of the plasma membrane to the cytoplasmic leaflet, these were as follows (the circumflex symbol ˆ marks the position of the stretch of bases removed). To remove the nucleotide stretch GTGGCCGTGATTGTG, corresponding to residues 158 to 162, primer sequences were 5′- CGGGACTCCACGˆGGTGCCTCCGTC -3′ (sense) and 5′- GACGGAGGCACCˆCGTGGAGTCCCG -3′ (antisense); this mutant was called TMA. To remove the nucleotide stretch GGGGGCTTCCTGGCT, corresponding to residues 167 to 171, primer sequences were 5′- CACCAAGATGACCACˆGACGGAGGCACCCA -3′ (sense) and 5′- TGGGTGCCTCCGTCˆGTGGTCATCTTGGTG -3′ (antisense); this mutant was called TMB. Finally, to remove the nucleotide stretch GTGCTGATGGTGGTC, corresponding to residues 176 to 180, primer sequences were 5′- TTTTCTCCTCACACACTTˆCAAGATGACCACAGCCAG -3′ (sense) and 5′- CTGGCTGTGGTCATCTTGˆAAGTGTGTGAGGAGAAAA -3′ (antisense); this mutant was called TMC. In these designs, we took into account the predicted TMD for β2, which corresponds to residues 160 to 180 in human β2 (23); see UniProtKB entry O60939. We also included Val-158 and Ala-159 due to their hydrophobic nature and because Val is considered typical of TMDs (36).

The generation of tail-minus β2-YFP, referred to here as 181X, has also been described. As the truncated protein was poorly expressed, a (Gly-Gly-Ser-Gly)_2_ linker between residue 181 and the YFP tag had to be added, which improved its expression levels (21). All constructs were verified by sequencing.

### Cell culture and transient transfection

MDCK cells II and transfectant derivatives were maintained in Minimum Essential Medium with Earl’s salts, supplemented with 10% FBS and 1% GlutaMAX (Gibco – Thermo Fisher Scientific); cells were validated and tested for contamination. Transfections were performed, following the manufacturer’s instructions, with Lipofectamine 2000 in Gibco Opti-MEM I reduced-serum medium (Invitrogen–Thermo Fisher Scientific), as described (24). To generate a fully polarized monolayer, cells were grown on 12 mm-diameter polycarbonate Transwell filters with 0.4 μm of pore size for a minimum of 2 days (Corning Inc., NY, US), as described (49). Non-polarized cells growing in wells were analyzed 1 or 2 days from transfection, depending on the experiment. As a control for transfections, we regularly used the pEGFP-N1 vector (Clontech, Takara Bio, Inc.).

### Generation of stable cell lines

Transfections were performed by calcium phosphate coprecipitation, as described (50), and single-cell clones were then selected with 200 μg ml^-1^ G418 (Sigma – Merck). Positive clones for YFP-tagged β2 mutants were identified visually using the appropriate filter under a fluorescence microscope and then confirmed by Western blot to β2 or GFP. Proper distribution of markers for cell surface (apical gp114 and basolateral p58) and tight junctions (Zonula Occludens-1, ZO-1) was then verified by immunofluorescence, ensuring normal polarity. The cell line expressing Na_V_1.5-YFP has been described (26).

### *In vitro* deglycosylation

Deglycosylation was performed in whole-cell lysates and the procedure used has been previously described in detail (24). Here, we have used endoglycosidase H (Endo H; NEB P0702; New England Biolabs) to discern between simple and complex *N*-glycosylation; Endo H cleaves *N*-glycans between the two *N*-acetylglucosamine moieties in the core region of the glycan chain on high-mannose and hybrid, but not complex, glycans.

### Antibodies

Some antibodies were provided by other researchers and have been previously described. These include the mouse monoclonal antibodies to gp114 (a cell adhesion molecule) and to p58 (the Na/K-ATPase β subunit) (51), and the rat monoclonal antibody against ZO-l (52). Commercially available mouse monoclonal antibodies used were those against the Na/K-ATPase α1 subunit (Abcam ab7671; Abcam, Cambridge), to GM130 (BD 610822; BD Transduction Laboratories), and to α-spectrin-1 (BioLegend, previously, Covance SIG-39700). Commercial rabbit polyclonal antibodies used were anti-β2 (Alomone ASC-007; Alomone Labs., Jerusalem, Israel), anti-GFP (Abcam ab290), anti-calnexin (Abcam ab75801), and anti-Na_V_1.5 (Proteintech 23016-1-AP). We also used a rabbit monoclonal antibody to E-cadherin (Cell signaling 3195–clone 24E10). Primary antibodies were used at 0.5 to 1 μg ml^-1^ of purified IgG for Western blot and about 5- to 10-fold more concentrated for immunofluorescence.

HRP-conjugated, secondary antibodies for Western blot were from Jackson ImmunoResearch (codes 111-035-003 and 115-035-003; Jackson ImmunoResearch Europe Ltd, Ely). The Alexa Fluor-labeled secondary antibodies for immunofluorescence (Molecular Probes – Thermo Fisher Scientific), all raised in goat, were the following: with excitation peak (Ex, expressed in nm) Ex 594, anti-mouse A-11005 and anti-rabbit A-11012; and with Ex 633, anti-mouse A-21050 and anti-rat A-21094.

### Sample preparation, Western blot, and quantitation of protein band intensity

Protein determination of cell lysates, sample preparation for SDS-PAGE and Western blot, and subsequent stripping for antibody reprobing, were done as described (21). To detect Na_V_1.5, cells were lysed in 1% SDS. Then, samples in Laemmli buffer were heated at 70 °C for 5 min. To run gels under non-reducing conditions, DTT was omitted and samples were not heated; in these experiments, lysates were prepared from cells growing in wells the day after transfection. Detection of blotted protein bands, by visualization in a ChemiDoc MP Imaging System (Bio-Rad), and band intensity quantitation, were done as previously (21). In all figures displayed, molecular weight markers are in kDa.

### Cell surface protein biotinylation

Surface protein biotinylation of cells growing in Transwells was done at 4 °C with EZ-Link Sulfo-NHS-SS-Biotin (Thermo Fisher Scientific 21441), a water-soluble and membrane impermeable reagent. The procedure followed has been described previously in detail (26, 53); the biotin reagent was used at 0.5 mg/ml DPBS^+^ at pH 8.0 and cells were labeled for 30 min at 4 °C. Biotinylation of cells growing in wells was done the day after transfection. Overnight pulldowns were obtained by incubation with NeutrAvidin (Thermo Fisher Scientific 53150). Pulldowns and input lysates were analyzed by Western blot and bands of interest were then quantified. The anti-GFP antibody was normally used to detect β2-YFP as well as APP-YFP. An identical procedure was followed to quantify surface protein from cells growing non-polarized in wells. To specifically estimate Na_V_1.5 arrival to the cell surface, we used the Image Lab software (Bio-Rad) and applied our previously described protocol (26). Briefly, we divided the percentage of Na_V_1.5 in pulldowns—corrected by the input load—by that of the plasma membrane loading control, then we balanced each variant of β2 with the number obtained in β2 WT. To examine apical endocytosis of β2, we proceeded as previously (21, 53).

### Confocal immunofluorescence microscopy and quantitative image analysis

Cells were grown polarized in Transwells, fixed with paraformaldehyde, and immunostained, essentially as described (53). Nuclei staining, with 4′,6-diamidino-2-phenylindole (DAPI), and mounting of the slides for fluorescence microscopy analysis, were done as described (53).

High magnification images were taken using a Nikon A1R confocal microscope (Nikon, Shinagawa, Tokyo, Japan) at a minimum pixel resolution of 512 × 512, using the NIS-Elements AR software, as described (54). We detected β2 by its fluorescent tag using the argon ion laser at the 488 nm excitation line. Images were exported to TIFF format and colocalization was assessed without image preprocessing using Fiji, the ImageJ-based package that includes the JACoP plug-in. Manders’ colocalization coefficients were then calculated to estimate the fraction of β2 present in a given subcellular compartment (26). The number of images or cells analyzed is indicated in the text or in the corresponding figure legend.

### Statistics

All experiments were performed a minimum of three times. Data are expressed as mean ± SD (error bars). Statistical significance was calculated by the two-tailed Student’s *t* test. Alternatively, we applied one-way ANOVA with Tukey's honest significant difference or Dunnett’s *post hoc* tests, using the R software for statistical computing (55), when differences among groups needed to be tested. *p* values are specified in the text, figures, or in figure legends.

## Data availability

All data are contained in the manuscript.

## Supporting information

This article contains supporting information (Figs. S1, S2, and S3).

## Conflict of interest

The authors declare that they have no conflicts of interest with the contents of this article.

## Figures and Tables

**Supplemental Figure S3:**