# Supplementary material for: Role of protein domains in trafficking and localization of the voltage-gated sodium channel β2 subunit
Source: J Biol Chem. 2024 Sep 28;300(11):107833. doi: 10.1016/j.jbc.2024.107833 (PMC11532958; doi:10.1016/j.jbc.2024.107833)
Supplement: Supplemental Figures S1–S3 legend [file mmc1.docx]

# Role of protein domains in trafficking and localization of the voltage-gated sodium channel β2 subunit

# Eric Cortada^1,2^, Ramon Brugada^1,2,3,4^, and Marcel Verges^1,2,3,^*

^1^Cardiovascular Genetics Group – Girona Biomedical Research Institute (IDIBGI-CERCA) – Edifici IDIBGI, 17190 Salt, Girona, Spain; ^2^Biomedical Research Networking Center on Cardiovascular Diseases (CIBERCV), Spain; ^3^Medical Sciences Dep. – Univ. of Girona Medical School, 17003 Girona, Spain; ^4^Cardiology Dep., Hosp. Josep Trueta – Univ. of Girona Med. School, Girona, Spain

Mailing address: C/ Doctor Castany, s/n – Edifici IDIBGI, 17190 Salt – Prov. of Girona – Spain

# Running title: β2 trafficking and localization

*Corresponding author: Marcel Verges, [mverges@gencardio.com](mailto:mverges@gencardio.com); [marcel.verges@udg.edu](mailto:marcel.verges@udg.edu); Tel +34 872 987087 Ext. 62

# Key words: cell culture, protein trafficking (Golgi), cell polarity, sodium channel, glycosylation, MDCK cells, voltage-gated sodium channel, *SCN2B*

# Supporting Information

## Supplementary Figure 1. The extracellular loop is important for complex N-glycosylation of β2 and needed for its efficient surface delivery.

Representative western blot, after cell surface biotinylation, showing reduced plasma membrane levels (Pulldown) and affected mature (complex) glycosylation (upper band) of all β2-YFP mutants on extracellular Cys residues transiently transfected in MDCK cells, except for C55A and, to a lesser extent, the triple C50/55/127A mutant. The fully unglycosylated mutant (N42,66,74Q; UNG) is also included for comparison of defective arrival to the cell surface. Lys indicates lysate.

## Supplementary Figure 2. Mutants on Cys affecting the Ig loop display intracellular aggregates.

Representative fluorescent microscope images of cells transiently expressing β2-YFP show, at one day from transfection, abundant intracellular aggregates of single and double mutants on Cys residues designed to disrupt the Ig loop, i.e., on C50 and C127, and the triple C50A/C55A/C127A mutant. Their pattern contrasts with the distribution of β2 WT, outlining the plasma membrane. Scale bar is 50 μm.

## Supplementary Figure 3. β3 remains intracellular, just like β1.

Representative western blots, after cell surface biotinylation, of polarized cells transiently expressing β3-GFP, β2-YFP, or β1-CFP. Neither β3 nor β1 are detected at the apical (API) or basolateral (BAS) surface, contrasting with apical enrichment of β2. Markers gp114 and Na/K-ATPase remain at their apical and basolateral domains in pulldowns, respectively, confirming proper cell polarity.
